# A Steel Ball Surface Quality Inspection Method Based on a Circumferential Eddy Current Array Sensor

**DOI:** 10.3390/s17071536

**Published:** 2017-07-01

**Authors:** Huayu Zhang, Fengqin Xie, Maoyong Cao, Mingming Zhong

**Affiliations:** 1 College of Mechanical and Electronic Engineering, Shandong University of Science and Technology, Qingdao 266590, China; zhanghuayu@sdust.edu.cn (H.Z.); Zhong_mm0820@sdust.edu.cn (M.Z.); 2 College of Transportation, Shandong University of Science and Technology, Qingdao 266590, China; 3 College of Electrical Engineering and Automation, Shandong University of Science and Technology, Qingdao 266590, China; caomy@sdust.edu.cn

**Keywords:** steel ball, circumferential eddy current array sensor, defects

## Abstract

To efficiently inspect surface defects on steel ball bearings, a new method based on a circumferential eddy current array (CECA) sensor was proposed here. The best probe configuration, in terms of the coil quality factor (Q-factor), magnetic field intensity, and induced eddy current density on the surface of a sample steel ball, was determined using 3-, 4-, 5-, and 6-coil probes, for analysis and comparison. The optimal lift-off from the measured steel ball, the number of probe coils, and the frequency of excitation current suitable for steel ball inspection were obtained. Using the resulting CECA sensor to inspect 46,126 steel balls showed a miss rate of ~0.02%. The sensor was inspected for surface defects as small as 0.05 mm in width and 0.1 mm in depth.

## 1. Introduction

Surface defects on steel ball bearings seriously affect the stability and service life of the bearing itself. To rapidly and accurately inspect and identify defective steel balls is fundamental to the development of the bearing industry and its application fields [1].

The Aviko series automatic inspector (Sorting Solutions Ltd., Bílina, Czech Republic) mainly uses a combination of vibration, photoelectric, and eddy current sensors to inspect the surface and subsurface qualities of steel balls [2]. A double-probe eddy current sensor has been used to scan and inspect steel ball surfaces, with two probes symmetrically arranged at 180 degrees on the testing mechanism outside of the steel ball [1]. An instrument composed of several electromagnets and a ring-shaped capacitive sensor has been designed, yielding an instrument capable of inspecting surface defects 0.05 mm in diameter and 0.01 mm in depth on a 4 mm diameter steel ball bearing [3]. A visual inspection technique has been employed, with visual features providing a rapid means of inspection of single balls [4].

Considering the above methods, the setup with a double-probe eddy current sensor includes an unfolding wheel, as the core component of the equipment, that is easily damaged and requires regular replacement, resulting in high maintenance costs [1]. In the multi-sensor Aviko system, the meridian unfolding mechanism of the steel ball surface is set up to reduce the complexity of the unfolding wheel, but the unfolding mechanism is still complex. The system, which employs a ring-shaped capacitive sensor, needs to operate under lapping oil, despite possessing a simple unfolding mechanism [3]. The specular nature of the bearing surface makes it difficult to inspect subsurface defects of steel balls [4].

**Figure 1:**
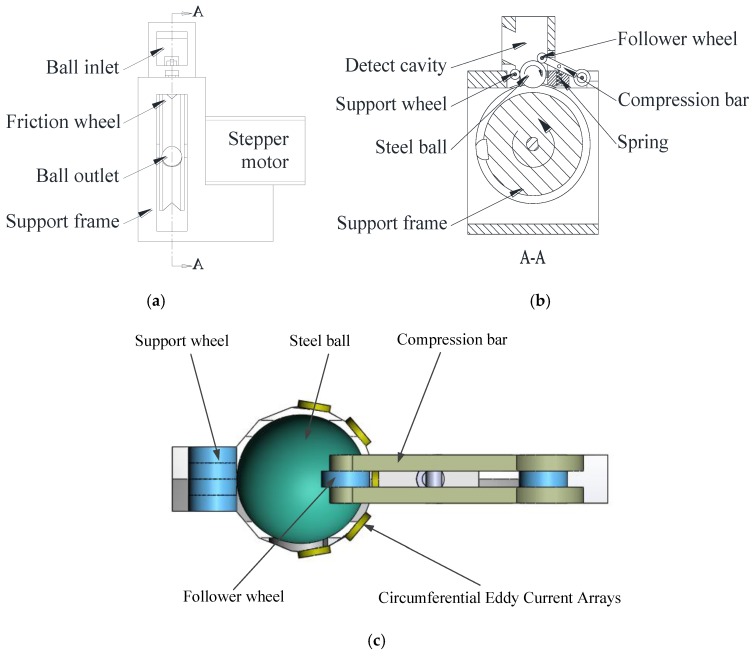
The unfolding mechanism of the steel ball. (**a**) The main view of the unfolding mechanism; (**b**) The cutaway view of the unfolding mechanism; (**c**) The top view of the unfolding mechanism.

In this paper, a new method for the quality inspection of steel ball surfaces was proposed based on a circumferential eddy current array (CECA) sensor. The method simplified the unfolding mechanism, reduced processing costs, and improved the equipment’s service life. The CECA sensor was composed of a number of probe coils in series, which were uniformly arranged on the latitudinal semicircle of the external support of the inspected steel ball (Figure 1). When the inspected steel ball was on a one-dimensional meridional movement driven by a frictional wheel, the steel ball surface was completely scanned and inspected by the CECA sensor. To inspect the ball surface completely, it was necessary to consider the cooperation of the CECA sensor and the unfolding mechanism of the steel ball. The following points concerning the best configuration of the probe coils were discussed:

(1) The theoretical basis of the probe operation.
(2) The appropriate number and arrangement forms of the probes.
(3) The quality factors (Q-factors) of four kinds of probe coil (3-, 4-, 5-, and 6-coil probes), and the frequency of coil excitation currents.
(4) The distributions of the eddy current density and magnetic field intensity on the ball surface induced by four kinds of eddy current array coils under the same direction excitation current, and determination of the optimal configuration of CECA sensors.

## 2. Unfolding Mechanism of the Steel Ball Surface 

The requirements for the surface defect inspection of steel balls were met by using a one-dimensional unfolding method to realize the full expansion of the steel ball. The unfolding mechanism of the steel ball is shown in Figure 1, which was mainly comprised of supporting, follower, and friction wheels, and a CECA sensor. The tested steel ball regularly entered into the inspection cavity through the ball inlet from a feeding system. 

The support wheel was used for supporting and positioning a ball, while the follower wheel was used for applying a force on the ball through a compression bar and a spring to prevent balls from bouncing up and down, and the friction wheel rotated the steel balls via a stepping motor. The CECA sensor collected the ball’s surface quality information, and then, the computer processed the information and judged whether the steel ball surface possessed defects.

In the inspection process, if the photoelectric sensor detected any position changes of the compression bar, this indicated that a ball had entered the inspection cavity; in this case, the friction wheel began to rotate and the CECA sensor was activated at the same time. After being completely inspected, the steel ball was moved out of the inspection cavity through the ball outlet to the sorting system, and the next ball entered. Repeating the above process, ball surface quality inspection was entirely automated.

## 3. Basic Principle of Eddy Current Testing for a Steel Ball

When excited by an alternating current, the probe coil generated alternating magnetic fields that induced eddy currents on the steel ball surface ahead of the coil. During inspection of ball surface quality, it was necessary to study the eddy current distribution on the ball.

**Figure 2:**
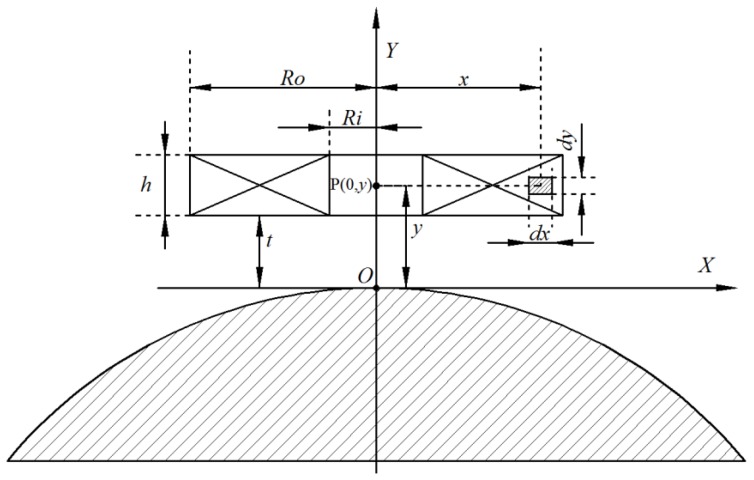
The model of eddy current testing.

The model for using the probe coil to inspect a ball surface is shown in Figure 2. If a micro unit in a section of coil was taken as *dx*∙*dy*, the spiral coil was considered a combination of many micro units, and the magnetic field produced by them was obtained by superposition. The current density of the micro unit was:

(1) $$\Delta i=\frac{NI}{\left(R_{o}-R_{i}\right)h}$$

where *N* is the coil turns, *I* is the total current flow through the coil, *h* is the coil thickness, *R_i_* is the inner coil radius, and *R_o_* is the coil outer radius*.*

According to Biot–Savart law, the magnetic induction intensity produced by the micro unit *dx*∙*dy* at point O (0, 0) is described in Equation (2):

(2) $$dB=\frac{\mu_{0}i}{2}\frac{x^{2}}{{\left(x^{2}+y^{2}\right)}^{3/2}}=\frac{\mu_{0}NI}{2\left(R_{b}-R_{a}\right)h}\frac{x^{2}}{{\left(x^{2}+y^{2}\right)}^{3/2}}dxdy$$

where $\mu_{0}=4\pi \times 10^{-7}$ (N·A^−2^) is the vacuum permeability, *x* is the radius of the micro unit, *y* is the distance between the micro unit and measured steel ball (lift-off), and *i* is the current through the micro unit *dx*∙*dy.*

The magnetic induction intensity produced by the coil at point *O* is:

(3) $$B_{O}=\int dB=\frac{\mu_{0}NI}{2\left(R_{o}-R_{i}\right)h}\left\{\left(t+h\right)\ln\frac{R_{o}+\sqrt{R_{o}^{2}+{\left(t+h\right)}^{2}}}{R_{i}+\sqrt{R_{i}^{2}+{\left(t+h\right)}^{2}}}-t\ln\frac{R_{o}+\sqrt{R_{o}^{2}+t^{2}}}{R_{i}+\sqrt{R_{i}^{2}+t^{2}}}\right\}$$

where $N=k\times (R_{o}-R_{i})\times h/\pi {R_{w}}^{2}$, $R_{w}$ is the radius of the enameled wire, and *k* is the filling factor. Taking *N* into consideration, Equation (3) can be modified as:

(4) $$B_{O}=\frac{k\mu_{0}I}{2\pi {R_{w}}^{2}}\left\{\left(t+h\right)\ln\frac{R_{o}+\sqrt{R_{o}^{2}+{\left(t+h\right)}^{2}}}{R_{i}+\sqrt{R_{i}^{2}+{\left(t+h\right)}^{2}}}-t\ln\frac{R_{o}+\sqrt{R_{o}^{2}+t^{2}}}{R_{i}+\sqrt{R_{i}^{2}+t^{2}}}\right\}$$

According to Equation (4), the magnetic induction intensity in the coil axis with four different dimensional parameters carrying 0.5 A current was calculated. The five coils with different parameters are listed in Table 1. 

**Figure 3:**
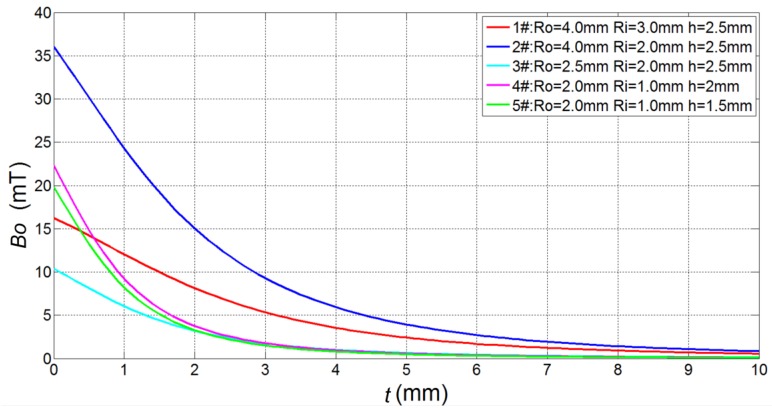
The relationship curves between magnetic induction intensity and lift-off.

Then, the *Bo–t* (the relationship between the magnetic induction intensity and lift-off) curves of the five different parameter coils were plotted (Figure 3). 

The magnetic induction intensity was observed to rapidly decrease with increasing distance *t* (lift-off, Figure 3)). For 1# coil, this decrease was 87% at *t* = 0.5 mm, 74% at *t* = 1 mm, and 22% at *t* = *R_o_*. For 2# coil, the magnetic induction intensity decreased to 84% at *t* = 0.5 mm, 67% at *t* = 1 mm, and 16% at *t* = *R_o_*. The intensity of 3# coil decreased to 78% at *t* = 0.5 mm, 58% at *t* = 1 mm, and 22% at *t* = *R_o_*. The intensity of 4# coil decreased to 66% at *t* = 0.5 mm, 41% at *t =* 1 mm, and 16% at *t* = *R_o_*. The intensity of 5# coil decreased to 67% at *t* = 0.5 mm, 41% at *t* = 1 mm, and 16% at *t* = *R_o_*. From the *Bo–t* of 4# and 5# coils, thickness showed little influence on the coil’s magnetic induction intensity. Therefore, the closer the distance between the ball and probe, the stronger the eddy current intensity, and thus the more favorable conditions were for the surface quality inspection of steel balls. On the other hand, the probe coil was arranged on the support frame outside the steel ball (Figure 1c) and the closer the steel ball was to the probe, the more difficult it was for the steel ball to enter the test chamber. A compromise scheme was adopted in which the inspection distance between the steel ball and probe was 0.5 mm, which was not only convenient for introducing a ball, but also ensured inspection accuracy of the surface quality.

## 4. Arrangement and Finite Element Analyses of the CECA Sensor

### 4.1. Arrangement of the CECA Sensor Probe Coil

The CECA coils needed to cover half of the ball circumference to inspect the steel ball surface completely, with each coil covering the distance of *C/2n* along the ball’s circumferential direction (Figure 1c). Consequently, the outer radius $R_{o}$ of each coil was:

(5) $$R_{o}=2Rsin\left(C/4nR\right)=2Rsin\left(\pi /2n\right)$$

where *C* is the steel ball circumference, *R* is the steel ball radius, and *n* is the number of CECA probe coils.

**Figure 4:**
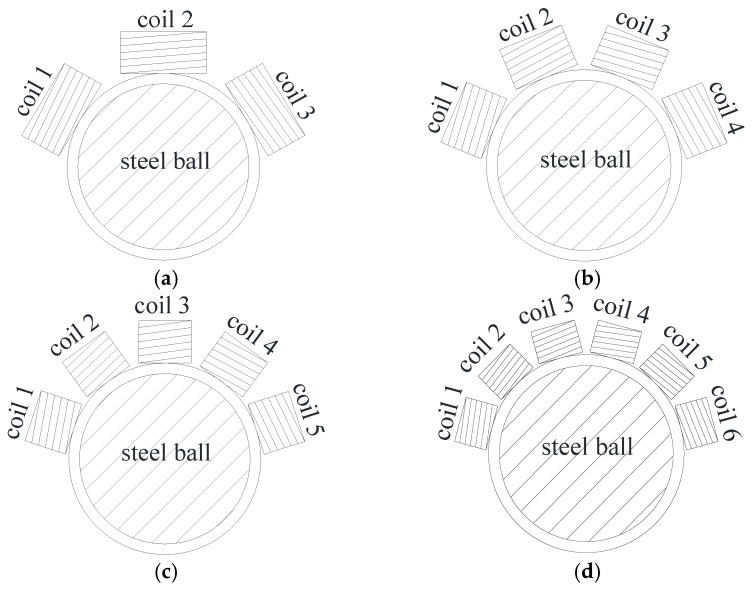
The arrangement of the multi-coil probe of the circumferential eddy current array (CECA) sensor. (**a**) 3-coil probe arrangement of CECA sensor; (**b**) 4-coil probe arrangement of CECA sensor; (**c**) 5-coil probe arrangement of CECA sensor; (**d**) 6-coil probe arrangement of CECA sensor.

For a steel ball 8 mm in diameter, if the number of probe coils was 2, the angle between the two coils was 90 degrees and the outer coil diameter was measured at 5.66 mm. Such a large coil outer diameter (relative to an 8 mm ball) reduced the sensor sensitivity and caused errant inspection of the ball surface. If the number of probe coils was 6, the balls were completely inspected, but the coil was difficult to mount on the support, so there was no need to use more than six coils. Therefore, the proper number of CECA probe coils was concluded to be 3 to 6. That is to say, there were four possibly useful probe coil arrangements: 3-, 4-, 5-, and 6-coil probes. For this series of probe forms, the angle between each coil was 60, 45, 36, and 30 degrees and the outer coil diameter was 4, 3, 2.5 and 2 mm, respectively (Figure 4).

### 4.2. The Coil’s Q-Factor

The Q-factor directly affected the inspection accuracy of the steel ball surface quality. When a coil was close to the steel ball, the power consumption of the coil increased, and the coil’s Q-factor reduced. The coil’s Q-factor, influenced by the measured clearance *t* (lift-off), was expressed as *Q(x)* [5]:

(6) Q(x)=2πfL(x)/R(x)

According to Equation (6), it was easy to see that the Q-factor was determined by the measured clearance *t,* the frequency of the coil’s excitation current, and the steel ball’s material. If the model of the steel ball was determined, under the same material properties and steel ball batch, the Q-factor of the probe coil was mainly determined by the measured clearance and frequency of the coil’s excitation current.

When the distance between the probe coil and steel ball was 0.5 mm, the inductance and resistance values were measured using an inductance, capacitance, and resistance (LCR) meter. In this work, an LCR IM3536 meter (Hioki E.E. Corp., Nagano, Japan) was used to measure the inductance and resistance. 

**Figure 5:**
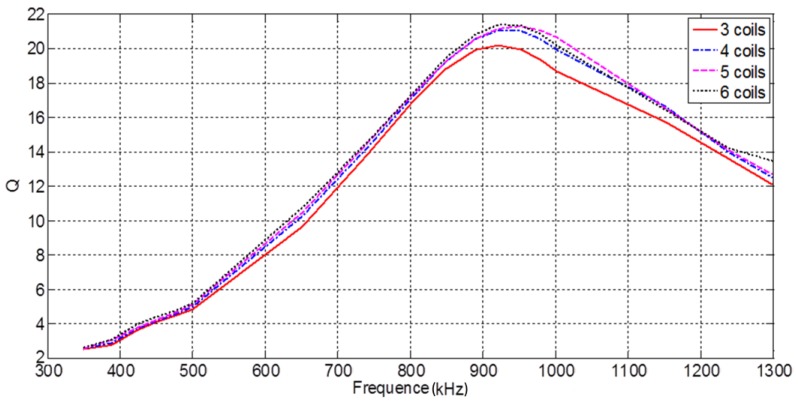
Q-factors of multi-coil probes with different excitation current frequencies.

The parameters for multi-coil probes in a CECA sensor under different arrangements are listed in Table 2. The four kinds of sensor setups (3-, 4-, 5-, and 6-coil probes) were all composed of copper wire, with a conductivity of 5.998 × 10^7^ S/m [6]. According to Equation (6), with the frequency of the coil’s excitation current variable from 350 kHz to 1.3 MHz, the corresponding Q-factor value was calculated and a fitting curve is plotted (Figure 5).

The Q-factor of a 3-coil probe in a CECA sensor reached a maximum of 20.1 at an excitation frequency of 920 kHz, but at 940 kHz, the Q-factor of 4-, 5-, and 6-coil probes reached the maximum, with values of 2121.5, and 21.6, respectively (Figure 5). From the above analysis, a coil excitation current of 940 kHz in a CECA sensor was found to be reasonable for 4-, 5-, and 6-coil probes.

## 5. The Finite Element Analyses in a CECA Sensor

Under the same excitation frequency and lift-off conditions, the stronger the eddy current density and magnetic field intensity on the ball surface, the lower the missed inspection rate for ball defects. The optimal number of coils in the CECA sensor was determined by analyzing the distribution of induced eddy current density and magnetic field intensity on steel balls under different coil arrangements with the finite element method.

Based on the above analysis of the probe coil’s Q-factor, the parameters of the multi-coils used in the CECA sensor and the measured ball are listed in Table 2, with the material of the steel ball being GCr15 (similar in grade to 52,100 in American, IIX15 in Russia, SUJ2 in Japan, En31 in the UK, and 100C6 in France). The relative magnetic permeability of GCr15 was $\mu_{r}\approx 1.0$ and the electrical conductivity 4.3478 × 10^6^ S/m [7].

**Figure 6:**
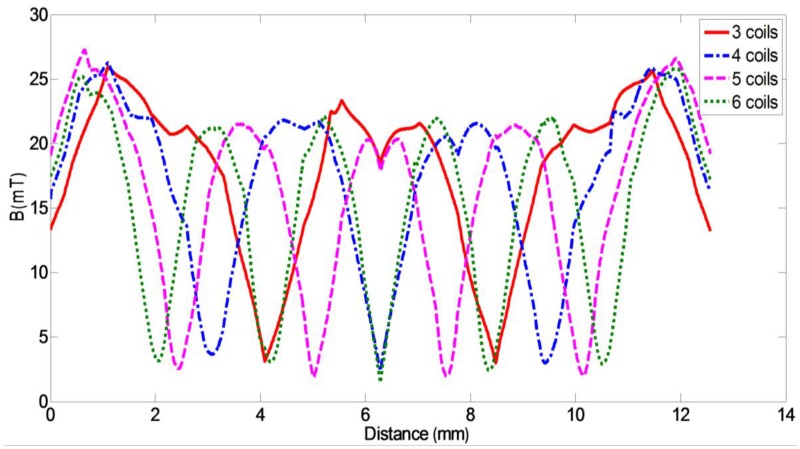
Distribution curves of induction intensities on a steel ball with different probe coils under the same direction excitation current.

The magnetic induction intensity distribution curves on a steel ball with different probe coil arrangements under the same direction excitation current (current, 0.5 A and frequency, 940 kHz) are shown in Figure 6. On the ball surface, for 3- and 4-coil probes, the magnetic induction intensity maximum amplitude was 26 mT, while the 5- and 6-coil probes were 28 and 25 mT, respectively. The distribution of magnetic induction intensity in 5- and 6-coil probes was observed to be more uniform than that of 3- and 4-coil probes.

**Figure 7:**
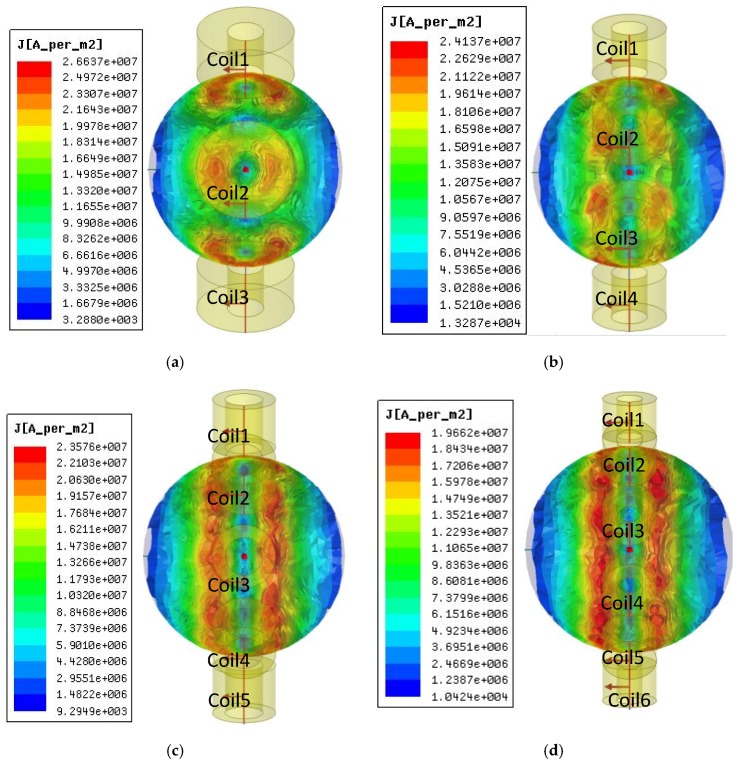
Eddy current density distributions of multi-coil probes in a CECA sensor on steel ball surfaces. The eddy current density distribution on a steel ball surface induced by (**a**) 3-coil probe; (**b**) 4-coil probe; (**c**) 5-coil probe; (**d**) 6-coil probe.

The eddy current distribution of the CECA sensor on the ball surface showed that the maximum eddy current density of the 3- and 4-coil probes was 2.6637 × 10^7^ and 2.4137 × 10^7^ A/m^2^, respectively, while that of the 5- and the 6-coil probes were 2.3576 × 10^7^ and 1.9662 × 10^7^ A/m^2^, respectively (Figure 7). Although the eddy currents induced by 3- and 4-coil probes were denser than that of the 5- and 6-coil probes, the distribution was not uniform. In particular, between adjacent coils, the minimum induction eddy current densities of the 3- and 4-coil probes were 3.288 × 10^3^ and 1.3287 × 10^4^ A/m^2^, respectively, which was a disadvantage for quality inspections of steel ball surfaces. The induced eddy current densities of 5- and 6-coil probes were uniform, but the induced eddy current density of the 5-coil probe was larger than that of the 6-coil probe. Therefore, the 5-coil probe was chosen as the optimal coil arrangement in this study. 

## 6. Experiments

The feasibility of using a CECA sensor to inspect the surface quality of a steel ball was verified by performing experiments on 8 mm GCr15 steel balls under the conditions of 0.5 mm lift-off and the same excitation current direction in each probe coil (current of 0.5 A and current frequency of 940 kHz). A 5-coil CECA sensor was used, in which the coils were uniformly distributed on the semicircle of the inspection chamber, and the parameters of the coils and steel ball are shown in Table 2. 

### 6.1. Inspection System

**Figure 8:**
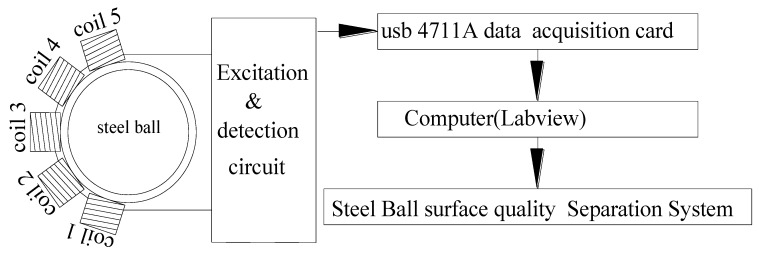
Block diagram of the experimental set-up for CECA sensor measurements.

**Figure 9:**
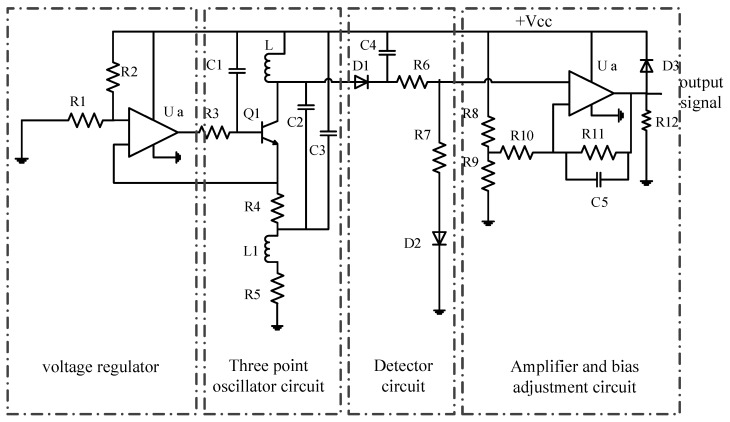
The CECA sensor inspection circuit.

A block diagram of the experimental set-up for CECA sensor measurements, which mainly included a CECA sensor, a data acquisition card of USB4711A (Advantech Co., Ltd., Milpitas, CA, USA), and a computer, is shown in Figure 8. The CECA sensor inspection circuit was composed of an oscillator, inspector, amplifier, and bias adjustment circuit (Figure 9). A capacitance-connecting three point-type oscillator was adopted as the oscillation circuit, using the CECA sensor coils as the inductive elements. The sensor’s Q-factor was changed into the amplitude and frequency of the high frequency carrier signal by the capacitive three-point oscillation circuit. The peak inspection circuit was used to inspect the output signal amplitude. After inspection, the output voltage signal was amplified and biased by the interface circuit, which helped in matching with the input voltage range of the data acquisition card. 

The voltage signal of the measured steel ball was compared with the setting threshold to judge whether the steel ball possessed a defect(s), which in turn controlled the actions of the sorting system, such that a defective steel ball was identified and sorted.

### 6.2. Results

**Figure 10:**
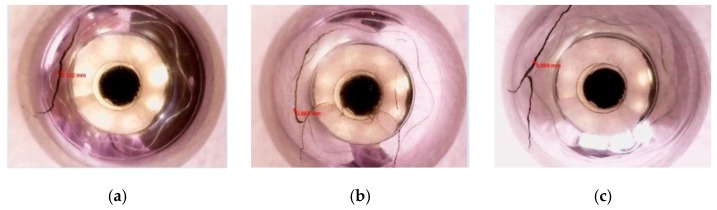
Samples of steel ball defects. (**a**) Linear cracks; (**b**) Arc cracks; (**c**) Cross cracks.

The common defects in steel balls include linear, arc, and cross cracks (Figure 10). The width of a linear crack is ~0.12 mm and depth ~0.5 mm; the width of an arc crack is ~0.05 mm and depth ~0.1 mm; the width of a cross crack is ~0.06 mm and depth ~0.9 mm.

Using the inspection system presented in this study to inspect the steel ball surface quality automatically, the testing experiments were completed in three steps. 

**Figure 11:**
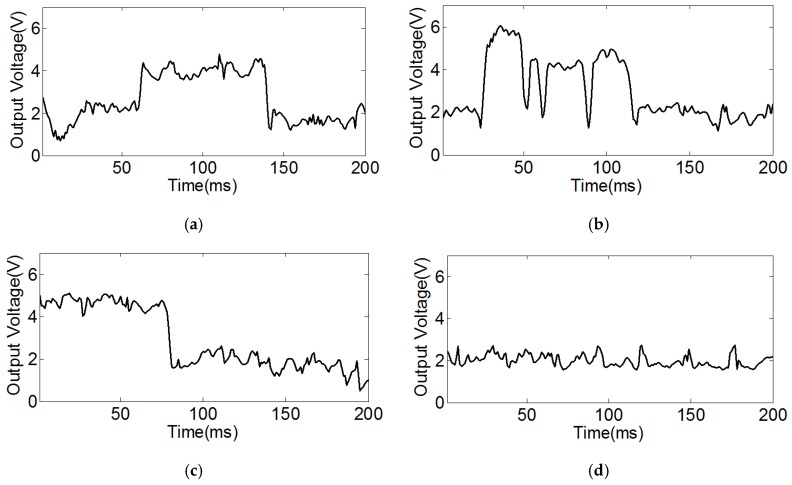
The inspection voltage curve. (**a**) The inspection curve of linear cracks; (**b**) The inspection curve of arc cracks; (**c**) The inspection curve of cross cracks; (**d**) The inspection curve of the steel ball without defect.

Step 1: Three steel balls with three kinds of standard defects and one steel ball without defect were placed into the inspection system. When the inspection threshold was set to 3.5 V, the results showed that there were obvious differences between the defective steel balls and the ball without defect (Figure 11).

Step 2: Twenty steel balls possessing the three kinds of standard defects were mixed into 980 qualified nondefective steel balls and then automatically sorted. The inspection results indicated 22 unqualified balls and 978 qualified balls. Then, the 22 unqualified balls were reexamined individually using a high-power microscope, and two of these balls were found to have been mistaken by the system for defective balls. Under the condition that all balls with defects were inspected and identified, a small number of qualified steel balls being mistaken for defective steel balls is acceptable.

Step 3: This inspection system was used to inspect 46,126 steel balls, randomly selected from seven batches of different quality steels. The process took 5.7 h and the inspection rate was 8092 balls/h. These 46,126 balls were then individually reexamined by a high power microscope. The results showed that only 10 defective steel balls were missed—a miss rate of 0.02%.

## 7. Conclusions

In this paper, a new method for inspecting steel ball surface defects based on a CECA sensor was proposed. This method decreased the dimension of the steel ball surface spreading mechanism and reduced the complexity of the unfolding wheel. Experimental results showed that the inspection miss rate was 0.02% and the inspection efficiency was 8092 balls/h. It was shown that this testing system effectively inspected and detected steel ball surface defects completely. 

## Figures and Tables

**Table 1:** Parameters of the probe coil.

Coil Number	The Outer Diameter *R_o_* (mm)	The Inner Diameter *R_i_* (mm)	The Thickness *h* (mm)
1#	4.0	3.0	2.5
2#	4.0	2.0	2.5
3#	2.5	2.0	2.5
4#	2.0	1.0	2.0
5#	2.0	1.0	1.5

**Table 2:** The parameters for multi-coil probes in a CECA sensor under different arrangements and parameters of the measured steel ball.

Parameters	3 Coils	4 Coils	5 Coils	6 Coils
The outer coil diameter (mm)	4	3	2.5	2
The inner coil diameter (mm)	1.5	1.5	1.5	1
Coil thickness (mm)	2	2	2	1.5
Copper wire diameter (mm)	0.06	0.06	0.06	0.06
Turn number of each coil (mm)	320	260	222	200
Steel ball diameter (mm)	8	8	8	8
Steel ball material quality	GCr15	GCr15	GCr15	GCr15
